# Standardising Care and Treatment of Transthyretin Amyloid Cardiomyopathy

**DOI:** 10.5334/gh.1275

**Published:** 2023-11-20

**Authors:** Marianna Fontana, Aldostefano Porcari, Philip N. Hawkins

**Affiliations:** 1 National Amyloidosis Centre, Division of Medicine, University College London, Royal Free Campus, Rowland Hill Street, London NW3 2PF, UK; 2 Centre for Diagnosis and Treatment of Cardiomyopathies, Cardiovascular Department, Azienda Sanitaria Universitaria Giuliano-Isontina (ASUGI), University of Trieste, Trieste 34149, IT; 3 European Reference Network for rare, low prevalence and complex diseases of the heart (ERN GUARD-Heart), Europe

**Keywords:** Cardiac Amyloidosis, Transthyretin, Diagnosis, Treatment, Prognosis

## Abstract

Transthyretin cardiac amyloidosis (ATTR-CA) has been traditionally considered a rare and inexorably fatal condition. ATTR-CA now is an increasingly recognised cause of heart failure and mortality worldwide with effective pharmacological treatments. Advances in non-invasive diagnosis, coupled with the development of effective treatments, have transformed the diagnosis of ATTR-CA, which is now possible without recourse to endomyocardial biopsy in around 70% of cases. Many patients are now diagnosed at an earlier stage. Echocardiography and cardiac magnetic resonance have enabled identification of patients with possible ATTR-CA and more accurate prognostic stratification. Therapies able to slow or halt ATTR-CA progression and increase survival are now available and there is also evidence that patients may benefit from specific conventional heart failure medications. A wide horizon of possibilities is unfolding and awaits discovery.

During the last decade, perception of cardiac ATTR amyloidosis has been radically transformed through major diagnostic advances in cardiovascular magnetic resonance (CMR) and bone tracer scintigraphy, and the development of treatments designed to ameliorate its natural history [1]. The flourishing science in this field has challenged traditional beliefs and has hugely promoted awareness and interest among doctors, patients and the pharmaceutical industry alike. As of today, five national or international societies of cardiology [2] have issued position statements or scientific statements on the diagnosis, management and treatment of amyloid cardiomyopathy in Europe (European Society of Cardiology [ESC] and German Cardiac Society [DGK]), Canada (Canadian Cardiovascular Society/Canadian Heart Failure Society [CCS/CHFS]), United States (American Heart Association [AHA]) and Japan (Japanese Circulation Society [JCS]). In this rapidly evolving scenario, it is essential to identify and, where possible, harmonise the different approaches to diagnosis and management of patients with transthyretin amyloid cardiomyopathy (ATTR-CM) adopted by expert opinion documents and position statements from single societies into an overarching perspective at international level.

In this issue of the Global Heart Journal, Brito et al. present the World Heart Federation Consensus on Transthyretin Amyloidosis Cardiomyopathy which provides a comprehensive review of ATTR-CM, prioritizing patients’ needs, including treatment accessibility, and the role of Patient Advocacy Groups, and highlighting the importance of multidisciplinary patient management, emphasizing the key role of specialized amyloidosis centers.

To achieve this aim, a panel of 18 expert clinicians specialized in ATTR amyloidosis from 13 countries was assembled along with a representative from the Amyloidosis Alliance, a patient advocacy group. Such a breadth of expertise is required since ATTR amyloidosis is highly heterogeneous with significant variation worldwide in terms of clinical phenotype at presentation, presence and consequence of the vast number of TTR gene mutations, diagnostic pathways, outcomes and treatment strategies. Identification of patients with early ATTR-CM has afforded the opportunity to broaden our understanding of its natural history and the factors influencing its clinical course such as the fibril type (ATTRwt vs. ATTRv amyloidosis), particular TTR gene mutations [3], age of onset, gender [4], severity of cardiac involvement [5] and, potentially, fibrils composition (fragmented vs. full-length TTR) [6].

Until recently, most patients with ATTR-CM were diagnosed with severe advancements already progressed throughout the disease, with characteristic echocardiographic features including severely increased wall thickness, restrictive LV filling pattern, atrial dilatation, and reduced ejection fraction [7]. However, the clinical phenotype of ATTR-CM at diagnosis has evolved with patients now being diagnosed earlier in the disease process. In a recent UK study, more than half of patients diagnosed from 2017 to 2021 were classified to have been diagnosed during earlier stages of the disease (National Amyloidosis Centre (NAC) ATTR stage I) which is associated with much better long-term survival [8]. Whilst ATTR amyloidosis might be perceived as a single entity, its accumulation underlies a spectrum of symptoms and differing clinical presentations [9]. Wild-type (non-hereditary) ATTR amyloidosis typically presents with cardiomyopathy, frequently with concurrent or prior carpal tunnel syndrome [10,11]; the clinical phenotype of variant (hereditary) ATTR amyloidosis varies greatly from predominant cardiomyopathy (ATTR-CM), to peripheral and or autonomic polyneuropathy (ATTR-PN), to a mixed phenotype (ATTR-CM + ATTR-PN) [12]. Importantly, phenotype can vary considerably even within a single family, including age of onset. Brito et al. underline that the presence of ATTR-PN should be systematically sought in all patients diagnosed with ATTRv amyloidosis as neurological involvement may develop in association with TTR variants widely held to only cause cardiomyopathy, since this can have implications for access to medicines and other aspects of treatment.

The use of CMR and repurposed bone scintigraphy for diagnosing ATTR-CM represents a milestone advance in the field, which has led to an exponential increase in the number of patients identified worldwide, and a much greater proportion being diagnosed at an earlier stage [13]. Brito et al. discuss some of the most challenging scenarios encountered in clinical practice such as the interpretation of incidental cardiac uptake in patients undergoing bone scintigraphy for other reasons. The presence of any myocardial uptake on bone scintigraphy is an abnormal finding that should prompt further investigation, however interpretation of a positive scan can be challenging in the absence of signs and symptoms of heart failure or in association with near normal echocardiographic or CMR findings. In this scenario, myocardial uptake could reflect currently incidental cardiac amyloid deposition, but early deposits may occur concomitantly with other cardiac pathologies, commonly for example with aortic stenosis, and may contribute to symptoms in such circumstances. Further research is required to elucidate the continuum from indolent amyloid deposits to overt amyloid cardiomyopathy and the associated clinical significance of the different stages in this very wide spectrum, both as sole pathologies and in association with others. As a note of caution, although three bone tracers have been validated for diagnosis of ATTR-CM, some clinically meaningful differences have been reported [14], which require further specific research. Multidisciplinary assessment, taking into account of all clinical, imaging and lab data is therefore paramount.

ATTR-CM is now treatable, making early diagnosis all the more important. Remarkably, a number of very different therapeutic strategies have been developed, with theoretical potential to be used together. Small molecule drugs that bind to circulating TTR, the liver-derived amyloid precursor protein, can enhance its stability with the aim of reducing its propensity to misfold and aggregate into ATTR amyloid fibrils. The TTR stabiliser tafamidis, was the first ever drug to be approved for any kind of amyloidosis; in the ATTR-ACT trial for ATTR-CM [15], treatment with tafamidis was associated with 30% reduction in all-cause mortality and 32% reduction in cardiovascular-related hospitalizations. The results from the ATTRibute-CM trial testing another stabilizer, acoramidis, have recently been presented and showed consistently positive results across all endpoints [16]. A second strategy, validated in the treatment of systemic amyloidosis generally, has been development of gene silencers to substantially reduce production of the TTR amyloid precursor protein. Inotersen, an antisense oligonucleotide, and patisiran, a small interfering RNA, suppress production of TTR by around 75–85% [17] and have been approved for hereditary ATTR-PN, and are currently in trial for ATTR-CM. Post hoc analysis of the seminal patisiran ATTR-PN trial suggested that greater knockdown of TTR was associated with better neurological outcomes [17], consistent with decades of clinical experience in AA and AL amyloidosis substantiating efforts to suppress production of the respective amyloid precursor proteins as much as possible. Encouragingly, recent real-world observations using CMR with extracellular volume (ECV) quantification have demonstrated regression of cardiac amyloid in some patients with ATTRv-CM who received patisiran treatment for a year, indicating that the balance of amyloid formation and slow natural clearance can be tipped towards the latter with adequate suppression of the former [18].

A quantum leap beyond gene silencing is permanent one-shot interference of the TTR gene by means of CRISPR-Cas9 (clustered regularly interspaced short palindromic repeats and associated Cas9 endonuclease) gene editing. Long applied in plant biology, current trials in ATTR amyloidosis represent its first in vivo use in humans. This treatment approach achieves near sustained up to 95% knockdown of TTR serum concentrations in patients with hereditary ATTR-PN [19], and it is now being tested in patients with ATTR-CM. Whilst very promising, neither TTR stabilising nor knockdown therapies have any direct impact on amyloid that is already present in the tissues, and therefore remains an unmet need for treatment that can enhance the otherwise very slow natural turnover of amyloid, especially for patients diagnosed at a late stage. A third treatment strategy, with this in mind, and which is now being hotly pursued, is the potential for antibody mediated elimination of amyloid, a strategy both encouraged and cautioned by analogous research in Alzheimer disease associated brain Abeta amyloid [20]. Excitingly, the plausibility for such an approach in ATTR amyloidosis has lately been supported by two different studies. A recently published phase 1 clinical trial of NI006, a recombinant human IgG1antibody that binds all non-soluble conformations of TTR, in 40 patients with ATTR-CM [21] suggested possible efficacy through decreases in serum cardiac biomarkers, cardiac uptake of bone tracers and myocardial ECV values, which would be consistent with a reduction in cardiac amyloid load. Further evidence suggesting a therapeutic role for anti-amyloid antibodies emerged from mechanistic investigations of three unique ATTR-CM patients in whom unprecedented spontaneous recovery occurred in association with near complete clearance of their cardiac amyloid deposits [22]. Circulating polyclonal IgG antibodies specific for ATTR amyloid were present in each case. Moreover, elimination of amyloid was accompanied by remodeling of their hearts back towards normal with regards to both structure and function, and without fibrosis. These findings compellingly suggest that ATTR-CM is a truly reversible disorder, bringing hope for patients even with advanced disease.

These remarkable advances in therapy for ATTR-CM have between them shed substantial light on the continuously dynamic nature of cardiac amyloidosis, i.e. the interplay between amyloid production and amyloid clearance [18,22,23], both of which may vary from patient to patient. A challenge lying ahead will be elucidating how and when these therapies may be used to best benefit the ever increasing ATTR-CM patient population.

## References

[1] Porcari A, Merlo M, Rapezzi C, Sinagra G. Transthyretin amyloid cardiomyopathy: An uncharted territory awaiting discovery. European Journal Internal Medicine. 2020; 82: 7–15. DOI: 10.1016/j.ejim.2020.09.025 PMC7534738 33032855

[2] Rapezzi C, Aimo A, Serenelli M, Barison A, Vergaro G, Passino C, et al. Critical Comparison of Documents From Scientific Societies on Cardiac Amyloidosis: JACC State-of-the-Art Review. Journal American College Cardiology. 2022; 79: 1288–1303. DOI: 10.1016/j.jacc.2022.01.036 35361352

[3] Porcari A, Razvi Y, Masi A, Patel R, Ioannou A, Rauf MU, et al. Prevalence, characteristics and outcomes of older patients with hereditary versus wild-type transthyretin amyloid cardiomyopathy. European Journal Heart Failure. 2023; 25: 515–524. DOI: 10.1002/ejhf.2776 36644836

[4] Patel RK, Ioannou A, Razvi Y, Chacko L, Venneri L, Bandera F, et al. Sex differences among patients with transthyretin amyloid cardiomyopathy – from diagnosis to prognosis. European Journal Heart Failure. 2022; 24: 2355–2363. DOI: 10.1002/ejhf.2646 PMC10087683 36575133

[5] Chacko L, Karia N, Venneri L, Bandera F, Passo BD, Buonamici L, et al. Progression of echocardiographic parameters and prognosis in transthyretin cardiac amyloidosis. European Journal Heart Failure. 2022; 24: 1700–1712. DOI: 10.1002/ejhf.2606 PMC10108569 35779241

[6] Porcari A, Fontana M, Gillmore JD. Transthyretin cardiac amyloidosis. Cardiovascular Research. 2023; 118: 3517–3535. DOI: 10.1093/cvr/cvac119 35929637 PMC9897687

[7] Knight DS, Zumbo G, Barcella W, Steeden JA, Muthurangu V, Martinez-Naharro A, et al. Cardiac Structural and Functional Consequences of Amyloid Deposition by Cardiac Magnetic Resonance and Echocardiography and Their Prognostic Roles. JACC Cardiovascular Imaging. 2019; 12: 823–833. DOI: 10.1016/j.jcmg.2018.02.016 29680336

[8] Ioannou A, Patel RK, Razvi Y, Porcari A, Sinagra G, Venneri L, et al. Impact of Earlier Diagnosis in Cardiac ATTR Amyloidosis Over the Course of 20 Years. Circulation. 2022; 146: 1657–1670. DOI: 10.1161/CIRCULATIONAHA.122.060852 36325894 PMC9698091

[9] Rapezzi C, Lorenzini M, Longhi S, Milandri A, Gagliardi C, Bartolomei I, et al. Cardiac amyloidosis: the great pretender. Heart Fail Rev. 2015; 20: 11–124. DOI: 10.1007/s10741-015-9480-0 25758359

[10] Porcari A, Pagura L, Longo F, Sfriso E, Barbati G, Murena L, et al. Prognostic significance of unexplained left ventricular hypertrophy in patients undergoing carpal tunnel surgery. ESC Heart Failure. 2022; 9: 751–760. DOI: 10.1002/ehf2.13606 34755478 PMC8787962

[11] Milandri A, Farioli A, Gagliardi C, Longhi S, Salvi F, Curti S, et al. Carpal tunnel syndrome in cardiac amyloidosis: implications for early diagnosis and prognostic role across the spectrum of aetiologies. European Journal Heart Failure. 2020; 22: 507–515. DOI: 10.1002/ejhf.1742 31975495

[12] Garcia-Pavia P, Rapezzi C, Adler Y, Arad M, Basso C, Brucato A, et al. Diagnosis and treatment of cardiac amyloidosis: a position statement of the ESC Working Group on Myocardial and Pericardial Diseases. European Heart Journal. 2021; 42: 1554–1568. DOI: 10.1093/eurheartj/ehab072 33825853 PMC8060056

[13] Gillmore JD, Maurer MS, Falk RH, Merlini G, Damy T, Dispenzieri A, et al. Nonbiopsy Diagnosis of Cardiac Transthyretin Amyloidosis. Circulation. 2016; 133: 2404–2412. DOI: 10.1161/CIRCULATIONAHA.116.021612 27143678

[14] Porcari A, Hutt DF, Grigore SF, Quigley A-M, Rowczenio D, Gilbertson J, et al. Comparison of different technetium-99 m-labelled bone tracers for imaging cardiac amyloidosis. European Journal Preventive Cardiology. 2022; 30(3): e4–e6. DOI: 10.1093/eurjpc/zwac237 36256685

[15] Maurer MS, Schwartz JH, Gundapaneni B, Elliott PM, Merlini G, Waddington-Cruz M, et al. Tafamidis Treatment for Patients with Transthyretin Amyloid Cardiomyopathy. New England Journal Medicine. 2018; 379: 1007–1016. DOI: 10.1056/NEJMoa1805689 30145929

[16] https://www.acc.org/Latest-in-Cardiology/Clinical-Trials/2023/08/24/02/29/attribute-cm.

[17] Zhang KW, Stockerl-Goldstein KE, Lenihan DJ. Emerging Therapeutics for the Treatment of Light Chain and Transthyretin Amyloidosis. Translational Science. 2019; 4: 438–448. DOI: 10.1016/j.jacbts.2019.02.002 PMC6609907 31312767

[18] Fontana M, Martinez-Naharro A, Chacko L, Rowczenio D, Gilbertson JA, Whelan CJ, et al. Reduction in CMR Derived Extracellular Volume With Patisiran Indicates Cardiac Amyloid Regression. JACC Cardiovascular Imaging. 2021; 14: 189–199. DOI: 10.1016/j.jcmg.2020.07.043 33129740

[19] Gillmore JD, Gane E, Taubel J, Kao J, Fontana M, Maitland ML, et al. CRISPR-Cas9 In Vivo Gene Editing for Transthyretin Amyloidosis. New England Journal Medicine. 2021; 385: 493–502. DOI: 10.1056/NEJMoa2107454 34215024

[20] Honig LS, Barakos J, Dhadda S, Kanekiyo M, Reyderman L, Irizarry M, et al. ARIA in patients treated with lecanemab (BAN2401) in a phase 2 study in early Alzheimer’s disease. Alzheimer’s Dementia. 2023; 9: e12377. DOI: 10.1002/trc2.12377 PMC10026083 36949897

[21] Garcia-Pavia P, aus dem Siepen F, Donal E, Lairez O, van der Meer P, Kristen AV, et al. Phase 1 Trial of Antibody NI006 for Depletion of Cardiac Transthyretin Amyloid. New England Journal Medicine. 2023; 389: 239–250. DOI: 10.1056/NEJMoa2303765 37212440

[22] Fontana M, Gilbertson J, Verona G, Riefolo M, Slamova I, Leone O, et al. Antibody-Associated Reversal of ATTR Amyloidosis–Related Cardiomyopathy. New England Journal Medicine. 2023; 388: 2199–2201. DOI: 10.1056/NEJMc2304584 37285532

[23] Martinez-Naharro A, Patel R, Kotecha T, Karia N, Ioannou A, Petrie A, et al. Cardiovascular magnetic resonance in light-chain amyloidosis to guide treatment. European Heart Journal. 2022; 43(45): 4722–4735. DOI: 10.1093/eurheartj/ehac363 36239754 PMC9712028

